# Integrative Analyses of Metabolome and Transcriptome Reveals Apocarotenoid and Flavonoid Biosynthesis During Saffron ( *Crocus sativus* L.) Stigmas Development

**DOI:** 10.1002/fsn3.70712

**Published:** 2025-08-01

**Authors:** Jing Chen, Xuting Xu, Shuhui Yang, Xiaodong Qian, Yuanyuan Tao, Guifen Zhou, Jing Li, Limin Xu, Liqin Li

**Affiliations:** ^1^ Huzhou Central Hospital Fifth School of Clinical Medicine of Zhejiang Chinese Medical University Huzhou China; ^2^ Huzhou Central Hospital Affiliated Central Hospital of Huzhou University Huzhou China; ^3^ TCM Key Laboratory Cultivation Base of Zhejiang Province for the Development and Clinical Transformation of Immunomodulatory Drugs Huzhou China; ^4^ Zhejiang Chinese Medical University Hangzhou China

**Keywords:** crocins, flavonoids, metabolome, saffron stigma development, transcriptome, WGCNA

## Abstract

Previous research has primarily concentrated on elucidating the structural and functional aspects of genes involved in crocin biosynthesis. However, the complex changes in metabolites that affect nutritional value and their regulatory mechanisms during the stigma development of saffron ( *Crocus sativus* L.) remain elusive. In this study, we delved into the functionality and complexity of the biosynthetic pathways for flavonoids and apocarotenoids in saffron, compounds that hold substantial nutritional significance. To explore the accumulation patterns and metabolic basis of apocarotenoid and flavonoid during saffron stigma development, widely targeted metabonomic and transcriptomic analyses in three (S1–S3) and four (S0–S3) developmental periods were conducted, respectively. The combined analysis of metabolism and transcriptome data showed that crocetin and flavonoids were mainly biosynthesized in the early red stage of stigma development (S1), and crocins were synthesized in the late red stage (S2). While the abscisic acid (ABA) biosynthesis pathway, a branch of crocetin metabolism, was particularly active at anthesis (S3), the synthesis of picrocrocin also increased during this stage. By employing weighted gene co‐expression network analysis (WGCNA), key modules and hub genes associated with flavonoids and apocarotenoid biosynthesis were successfully identified. The starch and sucrose metabolism, as well as plant hormone signaling, are important for the synthesis of secondary metabolites, according to the enrichment analysis of key modules and functional analysis of several hub genes (*G6Pep*, *UGE1*, *SBEI*, *IBR3*, *CKL2*, and *ARF15*) from co‐expression networks. In addition, a number of environmental stimuli were regarded as being significant, including flooding and red light. Our results provide new insights into the biosynthetic pathways and critical developmental stages that can be targeted for regulating the contents of secondary metabolites, ultimately enhancing the nutritional value and quality of saffron.

## Graphical abstract

During the development of saffron stigmas, crocetin and flavonoids were primarily synthesized in the early red stage (S1), while crocins were synthesized in the late red stage (S2). The biosynthesis pathways of ABA and picrocrocin were particularly active at anthesis (S3). S1 and S2 were critical periods for regulating the synthesis of important secondary metabolites. Starch and sucrose metabolism, plant hormone signaling, and environmental stimuli (e.g., flooding and red light) were crucial for the synthesis of secondary metabolites. Several hub genes from co‐expression networks, including *G6Pep, UGE1, SBEI, IBR3, CKL2,* and *ARF15,* played important roles in these processes.

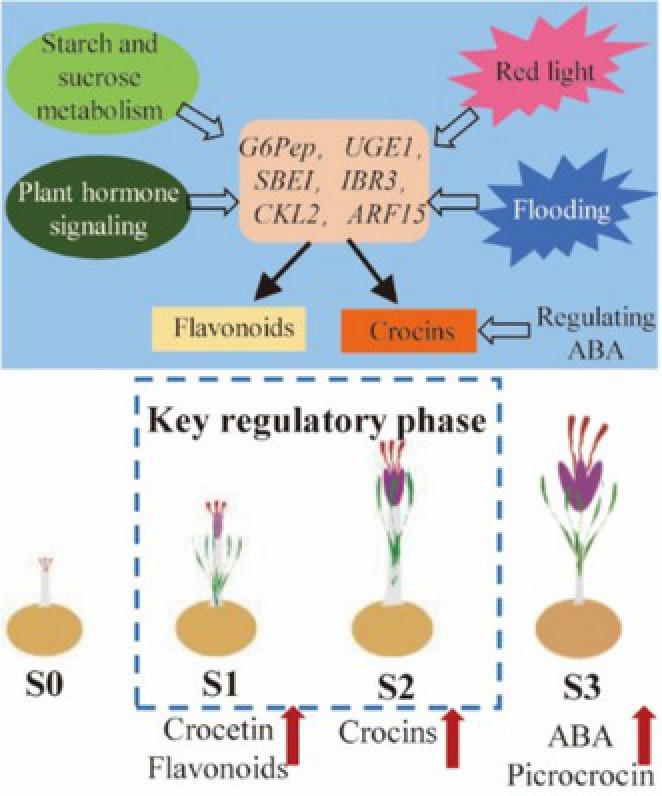

## 1 Introduction

*Crocus sativus* L., commonly known as saffron, is a perennial stemless herb related to the Iridaceae family, with Iran, China, Spain, Morocco, Italy, Greece, and India being some of its primary cultivation areas. In China, saffron occupies a significant role in traditional medicine and was officially listed in the Chinese Pharmacopeia in 1985, recognized by various names including “Xihonghua,” “Zanghonghua,” and “Fanhonghua” (National Pharmacopoeia Commission of China 1985). This herb has shown amazing therapeutic potential in treating a wide variety of ailments, such as cardiovascular diseases (Su et al. 2021), psychiatric disorders (Shafiee et al. 2017), neurodegenerative diseases (Bian et al. 2020), atherosclerosis (Kadoglou et al. 2021), learning and memory disorders (Hosseinzadeh et al. 2012), depression (Tóth et al. 2019), diabetes (Sani et al. 2022), and cancer (Xing et al. 2021). Recent research has revealed that saffron is a rich source of various pharmacologically active compounds, including carotenoids, flavonoids, terpenoids, amino acids, and alkaloids (Broadhead et al. 2016; Rahaiee et al. 2015). Crocin and crocetin are two important apocarotenoids isolated from saffron, which have been used as natural biomedicines (Zhao et al. 2021). Apocarotenoids (crocin, picrocrocin, and safranal), lutein, and abscisic acid (ABA) all trace their origins back to the common precursor, lycopene (Jain et al. 2016). Consequently, the biosynthesis of lutein or ABA might have an impact on the production yield of apocarotenoids. Saffron stigmas contain flavonoids as the second most abundant group of biologically active compounds, primarily composed of kaempferol glycoside derivatives (Mykhailenko et al. 2019), which demonstrate anticarcinogenic, anti‐inflammatory, antibacterial, antifungal, and antiprotozoal properties (Periferakis et al. 2022). Therefore, identifying and enhancing the content of these beneficial metabolites is crucial for maximizing saffron's therapeutic value in disease prevention and treatment.

Transcriptome sequencing stands as a crucial method for obtaining gene expression data in diverse organisms. Understanding the transcriptome in its entirety is crucial for elucidating the functional elements of the genome and for determining the molecular constituents of tissues and cells. In recent years, transcriptome sequencing has witnessed significant advancements and has been extensively employed to gain insights into the regulatory mechanisms underlying flower development, exemplified by studies on *Capsicum annuum* L. (Tang et al. 2023), pineapple (Wang et al. 2020), and chickpea (Singh et al. 2013). Additionally, the study of the transcriptome has significantly aided in uncovering and identifying genes that play a role in the biosynthesis of secondary metabolites (Pal et al. 2015; Zhou and Zhu 2020). The identification of transcripts associated with apocarotenoid biosynthesis within the saffron stigma, as well as the transcription factors implicated in secondary metabolism, has been achieved (Baba et al. 2015; Jain et al. 2016). However, it is difficult to get accurate gene annotation information because of the lack of saffron genome data. Metabolites, alongside genes and proteins, hold a central role in plant research. Metabolomics is an essential comparative technique for evaluating the total metabolite profiles in samples under various conditions or at different stages of development. The fluctuations in metabolite abundance reflect the chemical fluxes arising from a range of biochemical reactions, molecular mechanisms, and biological pathways. Given their proximity to the phenotype, metabolites are considered more representative of the physiological state of cells/organisms, offering a more direct glimpse into the cascading effects of environmental factors, gene expression, and regulatory processes (Astarita and Langridge 2013; Guijas et al. 2018).

In recent decades, combining metabolome profiling with transcriptome data has become a conventional method for identifying plant metabolites and elucidating their metabolic pathways (Luo 2015). For instance, metabolomics and transcriptomics were jointly utilized to investigate the variations in fruit flavor and carotenoid content between mature‐green and tree‐ripe stages of mango fruits, revealing insights into the regulatory mechanisms underlying β‐carotenoid biosynthesis and metabolism (Peng et al. 2022). Likewise, the research conducted by Xia et al. (2021) focused on examining the pigment accumulation and the mechanisms that regulate the color changes in petals throughout the flowering process of *L. japonica* . It is widely acknowledged that during the development of saffron stigmas, there are notable alterations in apocarotenoid levels and the gene expression profiles associated with apocarotenoid biosynthesis (Zhou et al. 2020). Besides, an integrated analysis of the carotenoid and transcriptome profiles during the stigma development process of saffron was conducted, revealing the significance of *CsPIF1*, *CsHY5*, and *CsPSY2* in regulating carotenoid synthesis (Thakuri et al. 2024). However, few studies have comprehensively explored the expression patterns of flavonoids, apocarotenoids, and other metabolites during stigma development in saffron through untargeted metabolomics and utilized transcriptome data to investigate the biosynthetic process and influencing factors of these metabolites.

The primary objective of the current study was to explore the main metabolites and pivotal regulatory genes involved in the development of saffron stigma. By clarifying the accumulation patterns of apocarotenoids and flavonoids, along with the expression profiles of their associated genes across different stigma development stages, we gained valuable insights that can guide future research on flavonoid and apocarotenoid biosynthesis pathways. To find the coexpression gene modules that were substantially related to the production of flavonoids and apocarotenoids, the weighted correlation network analysis (WGCNA) was used. By performing enrichment analysis and screening for hub genes, it is possible to elucidate the critical pathways that regulate the biosynthesis of secondary metabolites.

## 2 Materials and Methods

### 2.1 Plant Materials

Saffron plants from the identical clone line were grown at a research facility situated within the South Tai Lake Agricultural Park in Huzhou, with coordinates at longitude 120.6° E, latitude 30.52° N, and elevation 0 m. A two‐stage cultivation method was employed, where corms were planted in soil to facilitate outdoor growth, followed by indoor cultivation in a soil‐free environment. Stigmas were harvested at specific lengths of 5 mm (S0), 10 mm (S1), 20 mm (S2), and 35 mm (S3), respectively. Notably, the stigmas from Groups S0, S1, and S2 were harvested while still enclosed within the bud, whereas the stigmas from Group S3 were collected on the day of flowering. The stigmas gathered at each stage were combined and split into two distinct groups, one designated for metabolomic analysis and the other for transcriptomic analysis. It is worth mentioning that due to the limited availability of samples in Group S0, these stigmas were solely utilized for transcriptome analysis. For each group, three separate biological replicates were gathered for analysis. Post‐collection, the samples were immediately flash‐frozen in liquid nitrogen and then kept at −80°C for subsequent use.

### 2.2 Analysis of Crocins, Picrocrocin, ABA, and Total Flavonoids

A Jasper UPLC system (SCIEX) and an AB SCIEX Triple Quad 4500 mass spectrometer (SCIEX) equipped with an electrospray ionization (ESI) source were used to perform UPLC–MS/MS analysis. The UPLC separation was conducted on a chromatographic column with an inner diameter of 3.0 × 100 mm and a particle size of 2.6 μm (PFP 100 Å; Phenomenex), maintained at a temperature of 40°C. The mobile phases consisted of an aqueous amine acetate solution (0.5 mmol, phase A) and methanol (phase B), which were employed to separate crocin 1 and crocin 2. The flow rate for the separation was set at 0.5 mL min^−1^ under gradient elution conditions, and the injection volume was 5 μL. In multireaction monitoring (MRM) mode, crocin 1 and crocin 2 were detected by positive‐ion scanning. To determine the contents of picrocrocin, sample preparation was conducted following the ISO‐3632 procedure, with a proportional reduction in the amounts of saffron and solvent. Approximately 50 mg of saffron stigmas were carefully ground using a mortar. Subsequently, 10 mg of the resulting powder was placed into a 20 mL volumetric flask, to which 18 mL of distilled water was added to suspend the sample; this suspension was stirred magnetically in the dark for 1 h before being diluted to a final volume of 20 mL. The spectrophotometric measurement was performed on an appropriate aliquot of the aqueous extract after a 10‐fold dilution and filtration through a 0.45 μm cellulose filter (Merck Millipore Ltd., Bedford, Massachusetts, USA). The UV–vis spectra were obtained at 257 nm using a Bio‐Rad Smartspec Plus (Bio‐Rad, Hercules, California, USA) spectrophotometer, employing a 1 cm path length quartz cuvette and pure water for blank correction. The spectra were recorded with a resolution of 1 nm. The contents of ABA were calculated using the previously described UPLC–MS/MS method (Chen et al. 2024), and according to the manufacturer's procedure, total flavonoids were determined by Plant Flavonoids Colorimetric Assay Kit (Elabscience, Wuhan, China).

### 2.3 Sample Preparation and Metabolite Identification

To ensure the preservation of metabolic profiles, stigma samples at three different developmental stages (S1, S2, and S3) were collected and subjected to vacuum freeze‐drying for subsequent LC–MS widely targeted metabolomic analysis. Subsequently, these samples were pulverized using a grinding miller (MM 400, Retsch, Haan, Germany) operating at 30 Hz for a precise 1.5 min, ensuring a uniform powder consistency. Then, 100 mg of this powder was dissolved in 1.2 mL of an extraction buffer consisting of 70% ethanol, facilitating the efficient extraction of metabolites. The mixture was then allowed to extract overnight at a controlled temperature of 4°C, maximizing the yield of metabolites. Post‐extraction, the samples were subjected to centrifugation at 12,000 rpm for a duration of 10 min. Subsequently, the supernatant was passed through a filter prior to LC‐MS/MS analysis. The metabolite data were processed using Analyst 1.6.3 software (AB SCIEX, Ontario, Canada). Orthogonal Partial Least Squares Discriminant Analysis (OPLS‐DA) and Principal Component Analysis (PCA) were employed. Metabolites identified with a Variable Influence on Projection (VIP) score of 1 or higher and a fold change of at least 2 or no more than 0.5 were designated as differentially accumulated metabolites (DAMs).

### 2.4 RNA Extraction and Sequencing

Twelve libraries representing the four stigma development stages and the three replicates were constructed for transcriptome sequencing. RNA was extracted from the stigmas, with the enrichment of polyA‐tailed mRNA achieved through the use of magnetic beads coated with OligodT. Following this, the RNA was fragmented and reverse‐transcribed into cDNA. The double‐stranded cDNA, once purified, underwent end repair, was given an A‐tail, and then had sequencing adapters ligated to it. This comprehensive process ultimately yielded the desired library. Sequencing of the cDNA libraries was performed utilizing the DNBSEQ technology platform.

### 2.5 Raw Data Quality Control and Transcript Assembly

To ensure the integrity and quality of our data, the FastQC software (version 0.11.9) was employed to meticulously remove various contaminants and imperfections from the raw data. The process began with the removal of reads that had adapter sequence contamination, excessive poly‐N regions, or poor quality. Following this cleaning step, the high‐quality paired‐end reads were mapped against the reference genome (Xu et al. 2024) utilizing the STAR aligner (version 2.7.10). The raw data of reference genome and transcriptome sequencing in this study have been deposited by the authors in the Genome Sequence Archive (GSA) in the BIG Data Center, Beijing Institute of Genomics (BIG), Chinese Academy of Sciences, under accession number CRA007742 (http://bigd.big.ac.cn/gsa). Additionally, the assembled genome and gene structures of *Crocus sativus* have been deposited in Figshare (https://doi.org/10.6084/m9.figshare.21988667). Subsequently, the FeatureCounts software (v 2.0.1) was employed to count the reads that aligned to each gene. Ultimately, the expression levels of each gene were estimated in terms of fragments per kilobase of transcript per million base pairs (FPKM), taking into account the gene length and the number of reads aligned to it.

### 2.6 Construction of Weight Co‐Expression Network

Co‐expression networks were constructed using the WGCNA package (v 1.72.1) in R (Langfelder and Horvath 2008). A total of nine samples were included in this study, and the genes with FPKM values below 3 were filtered out. The soft thresholding power was determined by employing the pick‐soft threshold function. It was found that a threshold parameter β of 12 yielded a fitting curve close to 0.9, indicating a suitable soft threshold for our analysis. The adjacency matrix was converted into a topological overlap matrix (TOM) based on the β power. Genes exhibiting similar expression patterns were grouped into identical modules. Accumulations of nine metabolites obtained from the metabolomic analysis were designated as features. The corPvalueStudent function was utilized to perform a correlation analysis between each module and each metabolite. The similarity threshold for module assignment was set at 0.25, and a minimum count of 50 genes within each module was required to ensure meaningful comparisons. Finally, GS and MM were utilized to assess the correlations within the module.

### 2.7 Screening of DEGs in Four Stigma Development Stages

The DESeq2 R package was employed to perform differential expression analysis on the samples. *p* values obtained from this analysis were subsequently adjusted to control the false discovery rate (FDR) using the Benjamini and Hochberg method. Genes were considered differentially expressed if they had an adjusted *p* < 0.05 and an absolute log2‐fold change greater than 1. The Venn diagrams and bar charts were plotted by the R packages of VennDiagram (v 1.7.3) and ggplot2 (v 3.4.4), respectively. The clusterProfiler R package (v 4.6.2) was used for the Kyoto Encyclopedia of Genes and Genomes (KEGG) and Gene Ontology (GO) enrichment analyses.

### 2.8 Construction of Gene–Gene Correlation Networks and Identification of Hub Genes

Potential candidate genes were determined based on their intramodular connectivity (Lee et al. 2011), with the highest‐ranking 15 being designated as candidates. The networks of gene correlations were then visualized using Cytoscape (v3.7.1).

### 2.9 Real‐Time Quantitative PCR Validation

The cDNA was isolated from saffron stigmas at four distinct developmental stages. The subsequent experiment was conducted on an ABI 7500 Real‐time PCR system (Applied BioSystems, United States) employing SYBR Premix Ex Taq (Takara, Japan), following the manufacturer's guidelines. Tubulin served as the reference gene in this study. The relative expression levels were determined using the 2^−ΔΔCT^ method. Primers utilized in the study are detailed in Table S1.

### 2.10 Construction and Analysis of Recombinant Plasmids for Phenylalanine Ammonia‐Lyase ( *PAL* ), 4‐Coumarate: CoA Ligase ( *4CL* ), and Chalcone Synthase ( *CHS* )

The coding sequences for *PAL* (Csativus49639), *4CL* (Csativus01743), and *CHS* (Csativus50393) were obtained via amplification from cDNA templates. The primer sequences are listed in Table S2. Subsequently, these purified PCR products were inserted into the *pCAMBIA1302* binary vector, which had been linearized with NcoI, using the Gibson assembly method. The resulting recombinant plasmids, designated as *35S::PAL*, *35S::4CL*, and *35S::CHS*, were introduced into the *Agrobacterium tumefaciens* strain *GV3101* through electroporation. The Agrobacterium cells harboring these constructs were pelleted by centrifugation at 5000 g for 10 min and resuspended in an infiltration buffer (10 mM MgCl_2_, 10 mM MES, and 100 μM acetosyringone, pH 5.7) to achieve a final OD_600_ of 0.4–0.6. These suspensions were then infiltrated into the epidermal cells of 4‐week‐old Nicotiana benthamiana leaves. The leaves were harvested and subjected to analysis to determine the enzyme activities using the following assay kits: PAL Activity Assay Kit (Elabscience, China), 4CL Activity Assay Kit (mlBio, China), and ELISA Kit for CHS (mlBio, China).

## 3 Results

### 3.1 Phenotypic Variation and Metabolic Features

**FIGURE 1:**
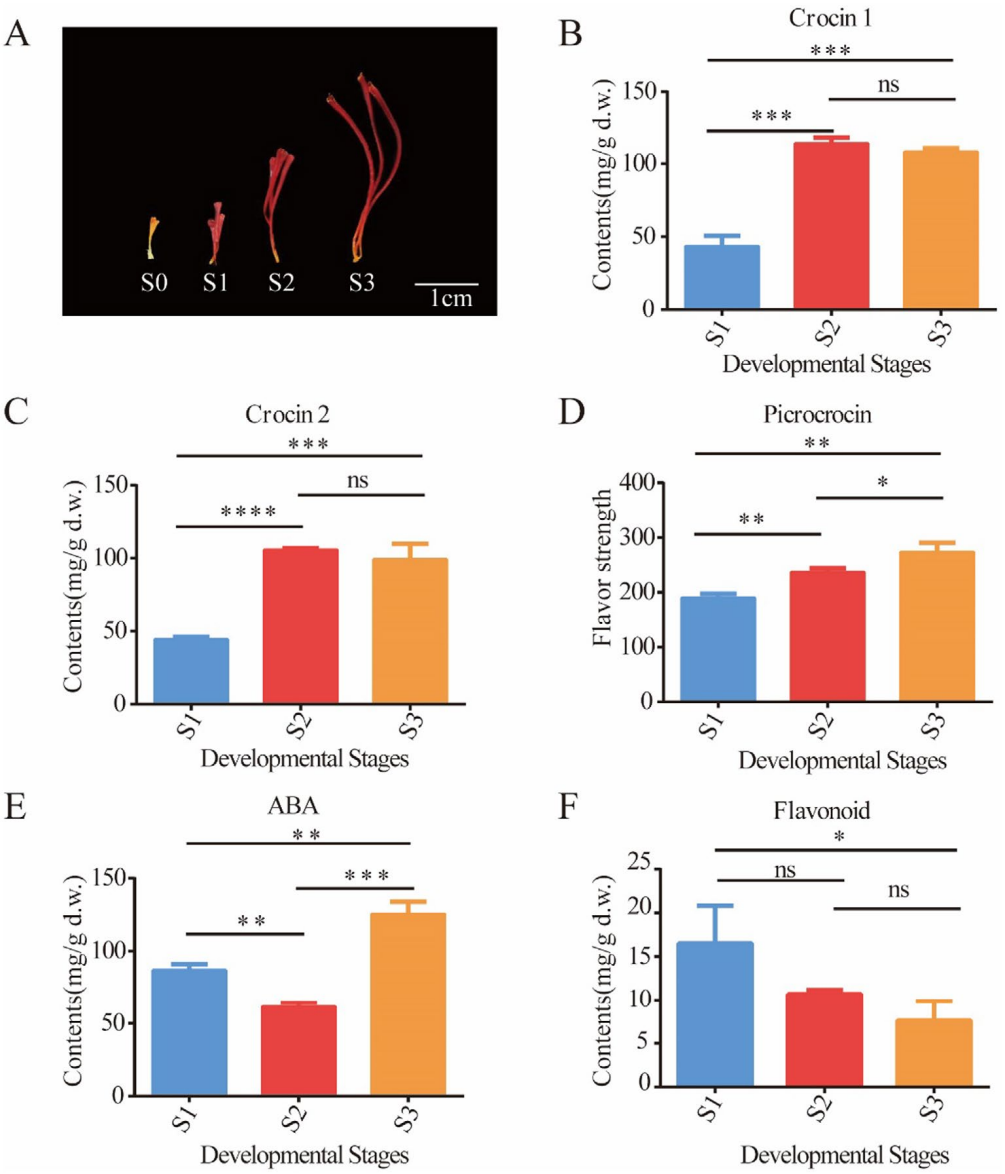
Physiological characteristics of saffron stigma throughout the flower development process. (A) Appearance of saffron stigma at S0 (5 mm, orange), S1 (10 mm, red), S2 (20 mm, red), and S3 (35 mm, scarlet, on the day of flowering). (B) Crocin 1 contents. (C) Crocin 2 contents. (D) Picrocrocin contents represented as flavor strength. (E) Abscisic acid (ABA) contents. (F) Flavonoid contents. Values (mean ± SD) were determined from three independent experiments (*n* = 3). Statistical significance was determined using an independent samples *t*‐test. *p < 0.05, **p < 0.01, ***p < 0.001, ****p < 0.0001.

The stigmas of saffron were utilized to explore the dynamic changes of metabolites during the developmental stage. The phenotype of the saffron stigma was investigated, as presented in Figure 1A. During this developmental process, the stigmas underwent a remarkable color transformation, shifting from orange to red and finally to scarlet. Due to the constraint in sample quantity, only the metabolic characteristics of saffron stigmas from stages 1 to 3 were analyzed (Figure 1B–F). The contents of crocin 1 and crocin 2 both witnessed a rapid increase from S1 to S2, followed by a slow decrease from S2 to S3. Given that ABA and crocin are both derived from the same precursor, lycopene, the contents of ABA in saffron stigmas from stages 1 to 3 were determined. The results demonstrated that, in contrast to the changing trend of crocin, the content of ABA decreased from S1 to S2 and then increased from S2 to S3. Picrocrocin levels, determined through ultraviolet spectrophotometry, showed a consistent increase. Flavonoids, which are the second‐most abundant category in saffron stigmas, were measured at various stages of saffron stigma development, and the results revealed a decline in total flavonoid levels in conjunction with the maturation of the stigmas.

### 3.2 Metabolome and Transcriptome Profiling of the Stigma Samples of Different Growth Stages

**FIGURE 2:**
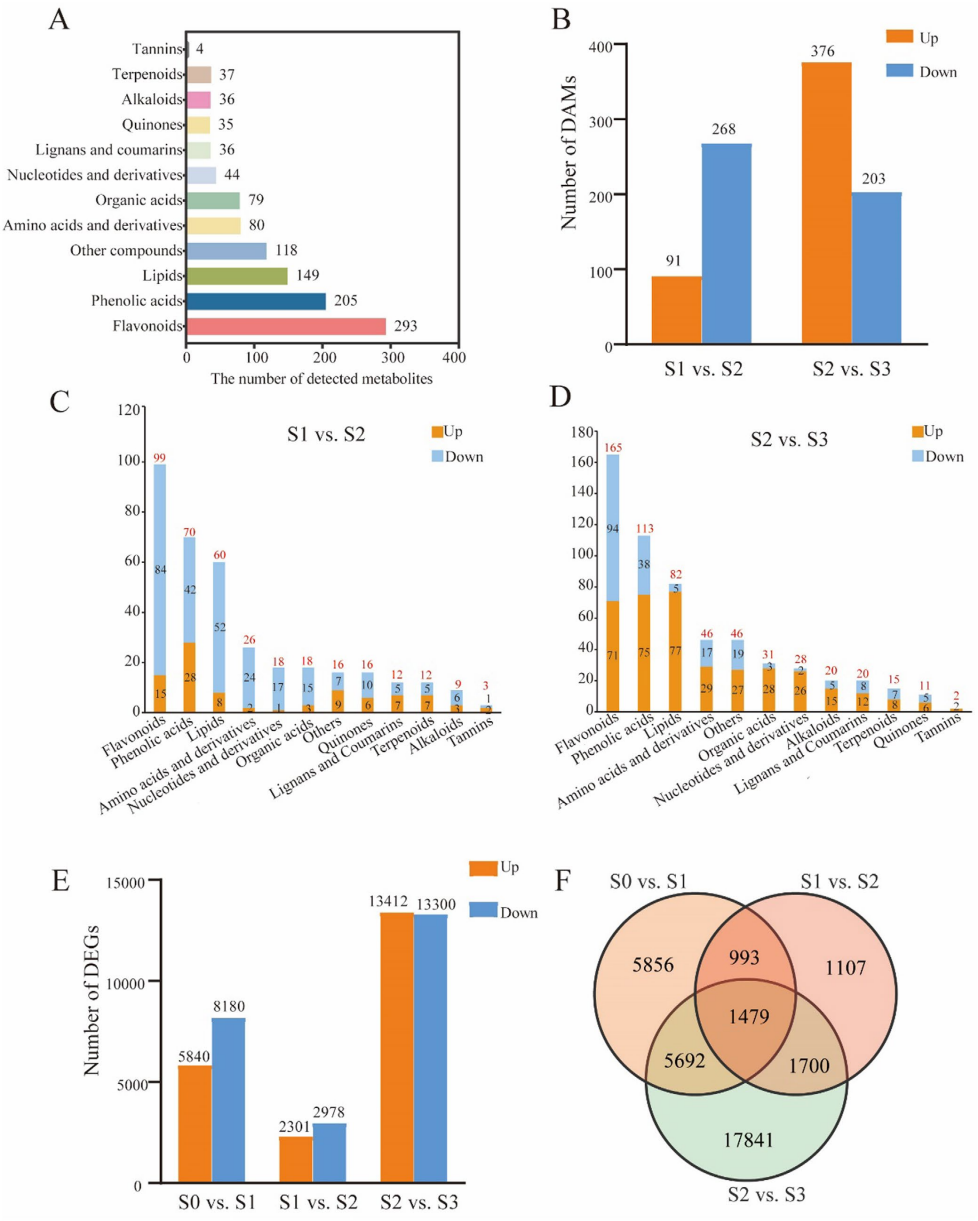
Preliminary analysis of metabolome data and transcriptome data. (A) The number of metabolites in 12 main categories. (B) Differentially accumulated metabolites (DAMs) in the comparisons of S1 vs. S2 and S2 vs. S3. (C) Number of DAMs up‐ and down‐regulated in different categories in the comparisons of S1 vs. S2. (D) Number of DAMs up‐ and down‐regulated in different categories in the comparisons of S2 vs. S3. (E) Differentially expressed genes (DEGs) in the comparisons of S1 vs. S2 and S2 vs. S3. (F) Venn diagram of DEGs during the three stigma development stages.

In order to understand the metabolite differences in the stigma development of saffron, a metabolomic comparison of saffron stigma at three developmental stages was conducted using high‐performance liquid chromatography‐mass spectrometry (HPLC‐MS/MS). After quality validation, a total of 1116 distinct metabolites were recognized and categorized into 42 different groups, as detailed in Table S3. Flavonoids, phenolic acids, lipids, other compounds, and amino acids and derivatives were among the top five most abundant categories among these metabolites, as illustrated in Figure 2A. DAMs were screened, and a total of 791 were identified among the three stages. Specifically, 359 and 579 DAMs were identified in the comparisons of S1 versus S2 and S2 versus S3, respectively. Comparing stage S1 with stage S2, there was an upregulation of 91 metabolites, accounting for 25.3%, and a downregulation of 268 metabolites, which constituted 74.7%. In the S2 versus S3 comparison, 376 metabolites (64.9%) were upregulated and 203 (35.1%) were downregulated (Figure 2B and Tables S4 and S5). In the comparative analysis of the metabolomes of S1 and S2, a greater number of secondary metabolites like flavonoids, phenolic acids, quinones, and alkaloids, as well as primary metabolites including lipids, amino acids and their derivatives, nucleotides and their derivatives, and organic acids are downregulated rather than up‐regulated. In the analysis contrasting S2 with S3, a higher number of metabolites exhibited up‐regulation compared to those that were down‐regulated, with the exception of flavonoids. (Figure 2C,D).

To visualize the overlap and uniqueness of DAMs across the different comparisons, a Venn diagram was generated. Twenty‐two metabolites were continuously up‐regulated during the three stages of pistil development. In particular, some metabolites related to cell structure formation, such as C (+)‐Sesaminol, lysoPE, and lysoPC, and those associated with energy metabolism and material transportation, such as picein and 6′‐O‐α‐D‐galactopyranosyl phlorigidoside. Additionally, a total of 39 metabolites showed a consistent decrease in expression across the developmental stages, with the majority being flavonoids, as illustrated in Figure S1A,B. To delve deeper into the biological processes potentially influenced by DAMs in the two comparative groups, a KEGG pathway analysis was conducted. It was observed that the downregulated metabolites in both the S1 versus S2 and S2 versus S3 comparisons were notably enriched in pathways associated with flavonoid biosynthesis, such as flavonoid biosynthesis, isoflavone biosynthesis, flavone and flavonol biosynthesis, and anthocyanin biosynthesis. This enrichment suggests that flavonoid biosynthesis metabolism gradually declines with pistil development. Furthermore, carotenoid biosynthesis was one of the top enriched KEGG terms in the comparison group of S1 versus S2, while in the comparison of S2 versus S3, pathways related to growth and development regulation and stress resistance response were enriched, such as butanoate metabolism, zeatin biosynthesis, 2‐Oxocarboxylic acid metabolism, and stilbenoid, diarylheptanoid, and gingerol biosynthesis (Figure S1C,F).

A comprehensive analysis of gene expression profiles was carried out throughout four distinct stigma development stages (S0, S1, S2, and S3), utilizing triplicate samples to construct a total of 12 cDNA libraries. Each individual sample yielded approximately 6 Gb of clean data, maintaining a Q30 base percentage exceeding 90% (Table S6). After filtering for differential expression genes (DEGs), the comparative analysis between S0 and S1 revealed that 5840 DEGs were upregulated and 8180 DEGs were downregulated. Between stages S1 and S2, the comparative analysis indicated an upregulation of 2301 genes and a downregulation of 2978 genes. Furthermore, when comparing S2–S3, there was a remarkable upregulation of 13,412 genes and downregulation of 13,300 genes (Figure 2E). Notably, it was observed that 1497 DEGs changed among all four development stages (Figure 2F).

### 3.3 Transcriptome and Metabolome Integrated Analysis of the Saffron Apocarotenoid Pathway

**FIGURE 3:**
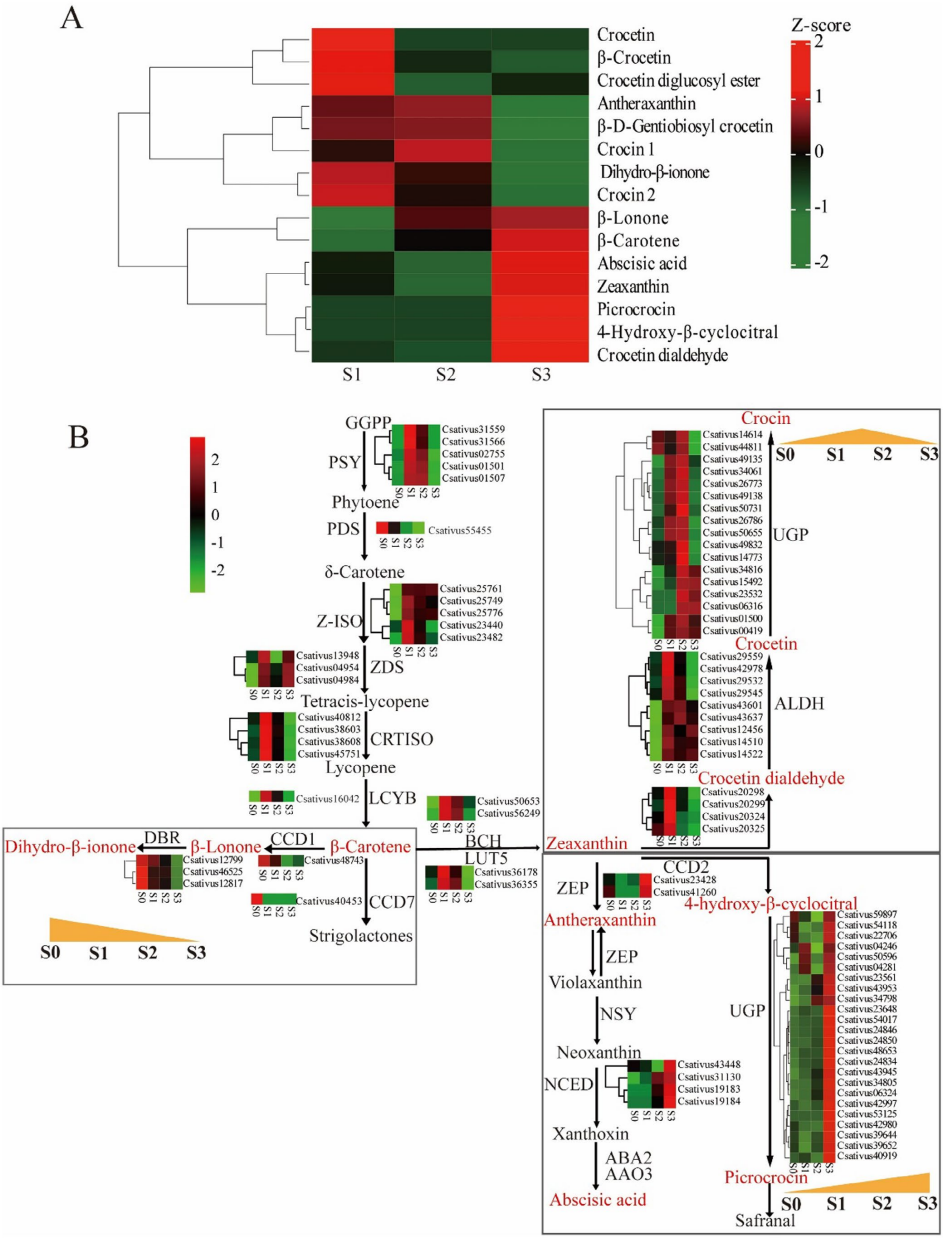
Metabolite and gene expression profiles associated with apocarotenoid biosynthesis in saffron stigmas. This pathway is constructed based on the Kyoto Encyclopedia of Genes and Genomes (KEGG) pathway and literary references. (A) Heat map of metabolites. (B) Expression of saffron apocarotenoid biosynthesis genes during saffron stigma development. The metabolites marked in red represent those that were detected in the metabolomic results, while the metabolites marked in black indicate those that were not detected. The framed areas highlight different patterns of metabolite accumulation.

The saffron stigmas contain three primary effective secondary metabolites, namely crocin, crocetin, and picrocrocin, all of which were detected in our metabolomics data. In addition, the intermediate metabolites in the saffron apocarotenoid pathway, such as zeaxanthin, 4‐hydroxy‐β‐cyclocitral, and crocetin dialdehyde, were identified. Besides, metabolites present on branching pathways, such as antheraxanthin, ABA, and dihydro‐β‐ionone, were also detected and analyzed. A heatmap was constructed to depict the varying accumulation patterns of metabolites throughout the developmental stages, as illustrated in Figure 3A. Notably, the downstream metabolites of crocin 1 and crocin 2 reached their highest accumulation levels in stages 2 and 1, respectively. Conversely, intermediate metabolites like β‐carotene, zeaxanthin, and crocetin dialdehyde had decreased levels in the early stages due to their conversion into downstream products. In stage 1, the crocetin accumulated to the highest extent. However, a high accumulation of ABA and picrocrocin was observed in stage 3. The analysis disclosed that the patterns of ABA and picrocrocin concentrations aligned with the earlier data shown in Figure 1, although minor discrepancies were noted in the levels of crocin. It was speculated that there were various types of crocin with similar structures, and it was extremely challenging for non‐target metabolomic approaches to completely separate the chromatographic peaks, thereby interfering with the quantitative outcomes.

The genes related to the apocarotenoid biosynthesis pathways were mapped, and their expression patterns were presented, as shown in Figure 3B. It was shown that the majority of genes involved in crocin biosynthesis exhibited upregulated expression in stage 1, including phytoene synthase (*PSY*), ζ‐carotene isomerase (*Z‐ISO*), ζ‐carotene desaturase (*ZDS*), carotene cis–trans isomerase (*CRTISO*), lycopene β‐cyclase (*LCYB*), β‐carotene hydroxylase (*BCH*), β‐ring hydroxylase (*LUT5*), carotenoid cleavage dioxygenase 2 (*CCD2*), and acetaldehyde dehydrogenase (*ALDH*). Consequently, crocetin accumulated to the highest extent in this stage. However, the expression level of the UDP–glucose pyrophosphorylase gene (*UGP*) did not reach its maximum until stage 2, causing the downstream metabolite crocin to have the highest content at this stage. Moreover, the expression level of *UGP* in the picrocrocin synthesis pathway reached its maximum in stage 3, leading to the greatest accumulation of picrocrocin at this stage. Interestingly, the genes associated with the biosynthesis of ABA, particularly zeaxanthin epoxidase (*ZEP*) and 9‐cis‐epoxycarotenoid dioxygenase (*NCED*), exhibited increased expression during stage 3, which coincides with the elevated levels of ABA detected at this developmental phase. Finally, the genes coding for carotenoid cleavage dioxygenase 1 (CCD1) and carotenoid cleavage dioxygenase 7 (CCD7), which are respectively involved in the biosynthesis of dihydro‐β‐lonone and strigolactone, were observed to be upregulated in stage 0. This observation suggests that these metabolites accumulate highest during the initial stages of saffron stigma development.

### 3.4 Transcriptome and Metabolome Integrated Analysis of the Flavonoid Pathway

**FIGURE 4:**
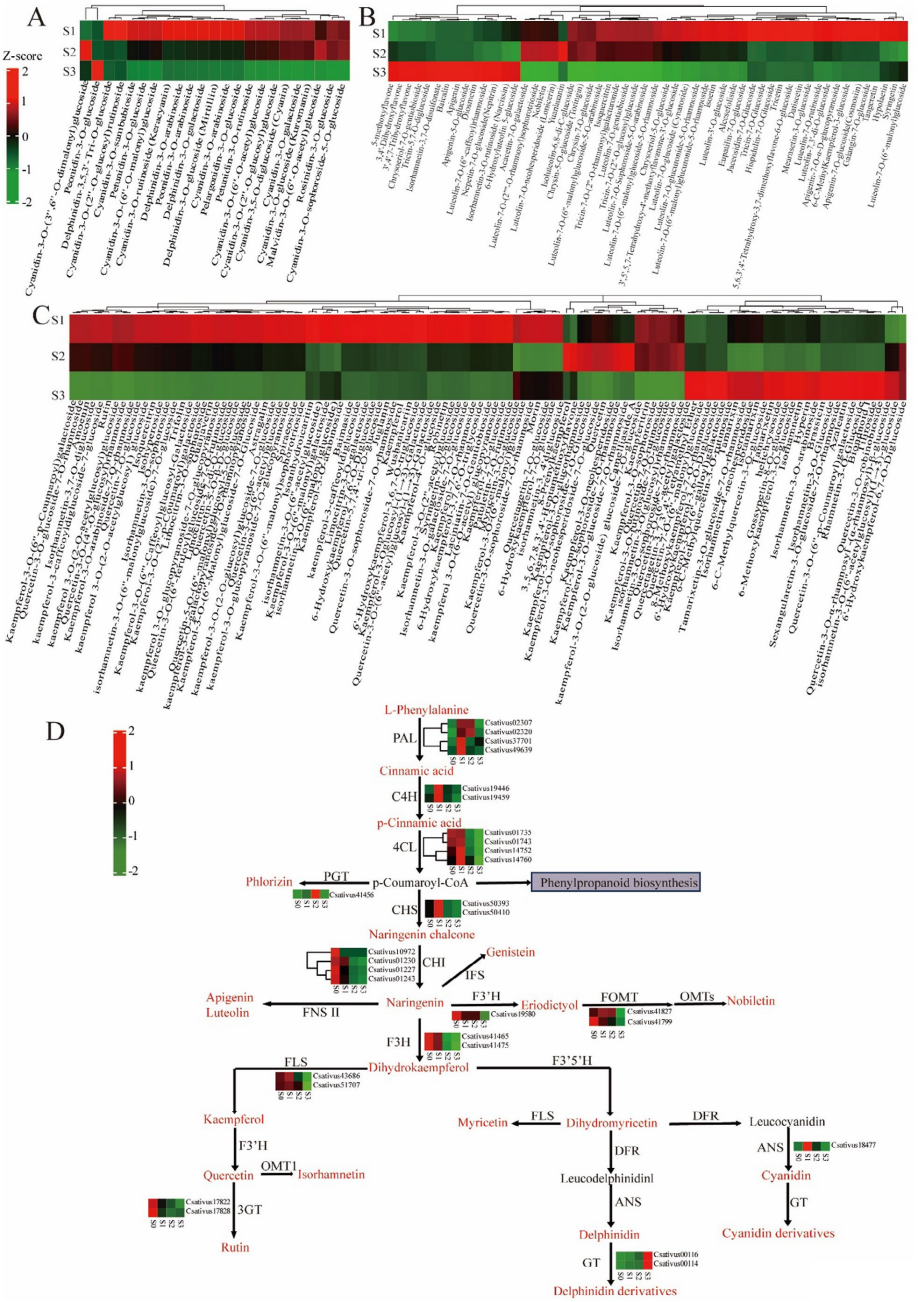
Flavonoid levels and heat map of flavonoid biosynthesis genes. This pathway is constructed based on the KEGG pathway and literary references. (A) Heat map of anthocyanins. (B) Heat map of flavones. (C) Heat map of flavonols. (D) Expression of flavonoid synthesis genes during saffron stigma development. The metabolites marked in red represent those that were detected in the metabolomic results, while the metabolites marked in black indicate those that were not detected.

Flavonoids play diverse and crucial roles in the developmental and adaptive processes of plants. Given the extensive variations observed in flavonoid profiles across the saffron stigma during three distinct growth stages, we conducted a comprehensive analysis to investigate the enrichment and clustering patterns of these compounds. A total of 293 flavonoids were successfully identified in the saffron stigma samples collected from these three growth stages. Ten different groups were used to systematically classify these flavonoids: flavones, flavonols, anthocyanidins, flavonoid carbonoside, isoflavones, flavanones, chalcones, flavanonols, dihydroisoflavones, and flavanols. The accumulation patterns of flavonoids revealed three distinct clusters that closely corresponded to the varying developmental stages of the saffron stigma. Notably, the majority of flavones, flavonols, and anthocyanidins exhibited high accumulation in the early stages of saffron stigma development (Figure 4A–C), which is in line with the total flavonoid contents shown in Figure 1F.

In an effort to probe the varying accumulation patterns of flavones, flavonols, and anthocyanidins throughout the three developmental phases of saffron stigmas, we combined the data of DEGs and DAMs associated with the flavonoid biosynthesis pathway, as outlined in map00491. The transcriptomic data of the saffron stigma showed distinct expression patterns among the four developmental stages for the structural genes crucial for flavonoid biosynthesis. To elaborate, the genes associated with the initial stages of the enzymatic reactions, such as *PAL*, cinnamate 4‐hydroxylase (*C4H*), *4CL*, and *CHS*, were predominantly upregulated in stage 1. Moreover, flavonoid O‐methyltransferase (*FOMT*), flavonol synthase (*FLS*), and anthocyanidin synthase (*ANS*) showed a general trend of upregulation in stage 1. Other genes, such as chalcone isomerase (*CHI*), flavanone 3‐hydroxylase (*F3H*), flavanone 3′‐hydroxylase (*F3*′*H*), and 3‐glucosyltransferase (*3GT*) exhibited significant upregulation in stage 0 (Figure 4D). A substantial number of genes that participate in the flavonoid biosynthetic pathway exhibited upregulation during the early phases of saffron stigma development, aligning with the observed accumulation patterns of flavonoids. This result not only expounds the accumulation pattern of flavonoids but also specifies the roles of these genes in regulating the flavonoid biosynthesis of saffron stigma. To verify the functions of the key genes involved in the early stages of flavonoid biosynthesis, we selected three genes, *PAL*, *CHS*, and *4CL*, and conducted transient expression experiments in tobacco. The results indicated that the enzyme activities of PAL and 4CL were significantly increased in the tobacco plants with high expression. Although the activity of CHS also increased, the difference was not statistically significant (Figure S2).

### 3.5 qRT‐PCR Analysis

**FIGURE 5:**
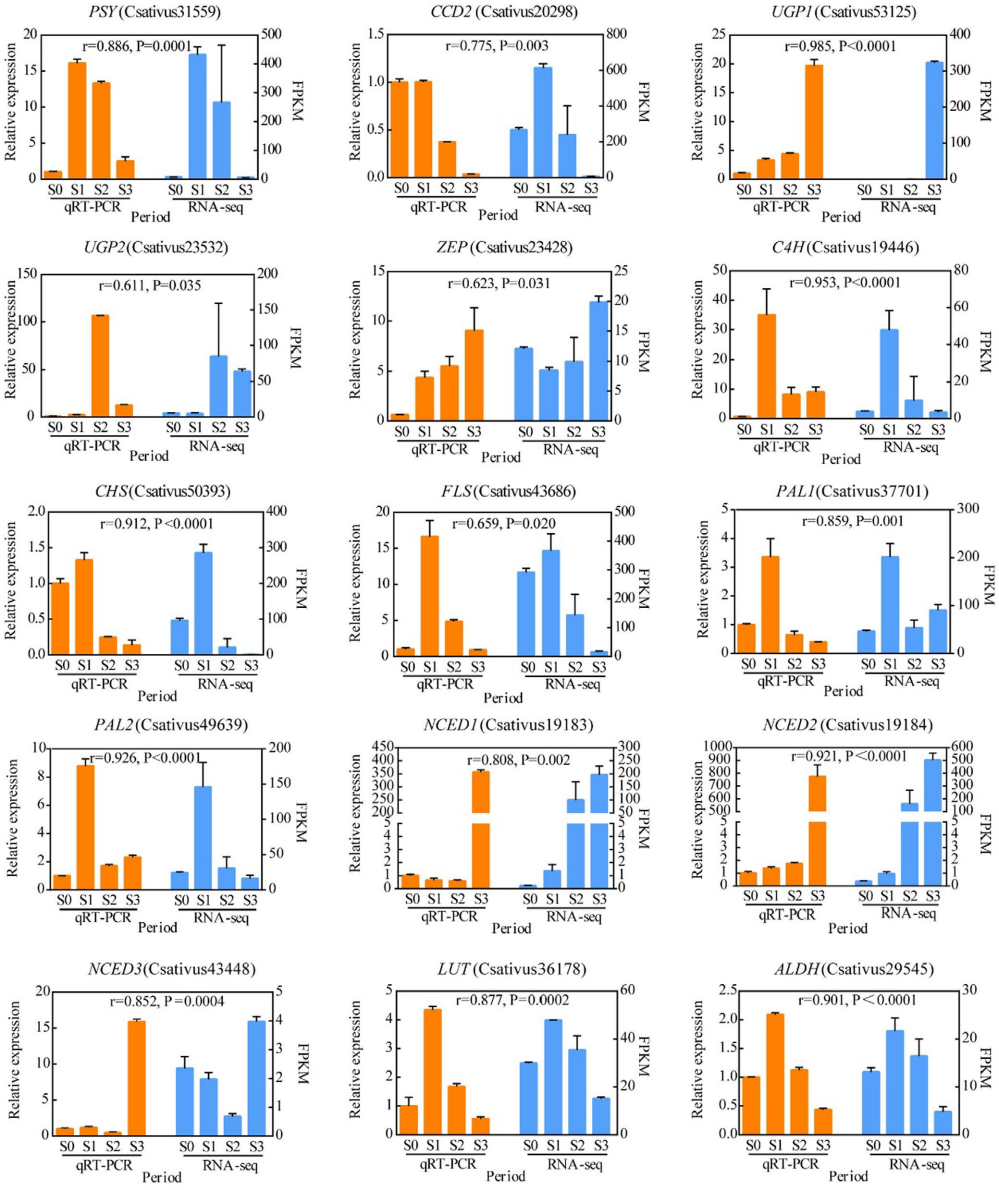
Relative expression of 15 genes involved in apocarotenoid and flavonoid biosynthesis during saffron stigma development. All data are presented as the mean of three biological replicates, and error bars represent standard deviation. The Pearson correlation coefficient is expressed as *r*.

To confirm the accuracy of the RNASeq data, 15 genes in the apocarotenoid and flavonoid biosynthesis pathway were selected. Additionally, qRT‐PCR was performed to evaluate the expression levels during the four developmental stages of the saffron stigma (Figure 5). The correlation coefficients were determined by comparing the transcriptome data with the real‐time fluorescent quantitative data for these genes. The results indicated that the expression trends of genes were highly correlated with the RNA‐Seq results, and their variation patterns were largely consistent. Therefore, based on these results, the reliability and reproducibility of the transcriptome data were verified.

### 3.6 Identification of Coexpressed Gene Networks and Key Candidates

**FIGURE 6:**
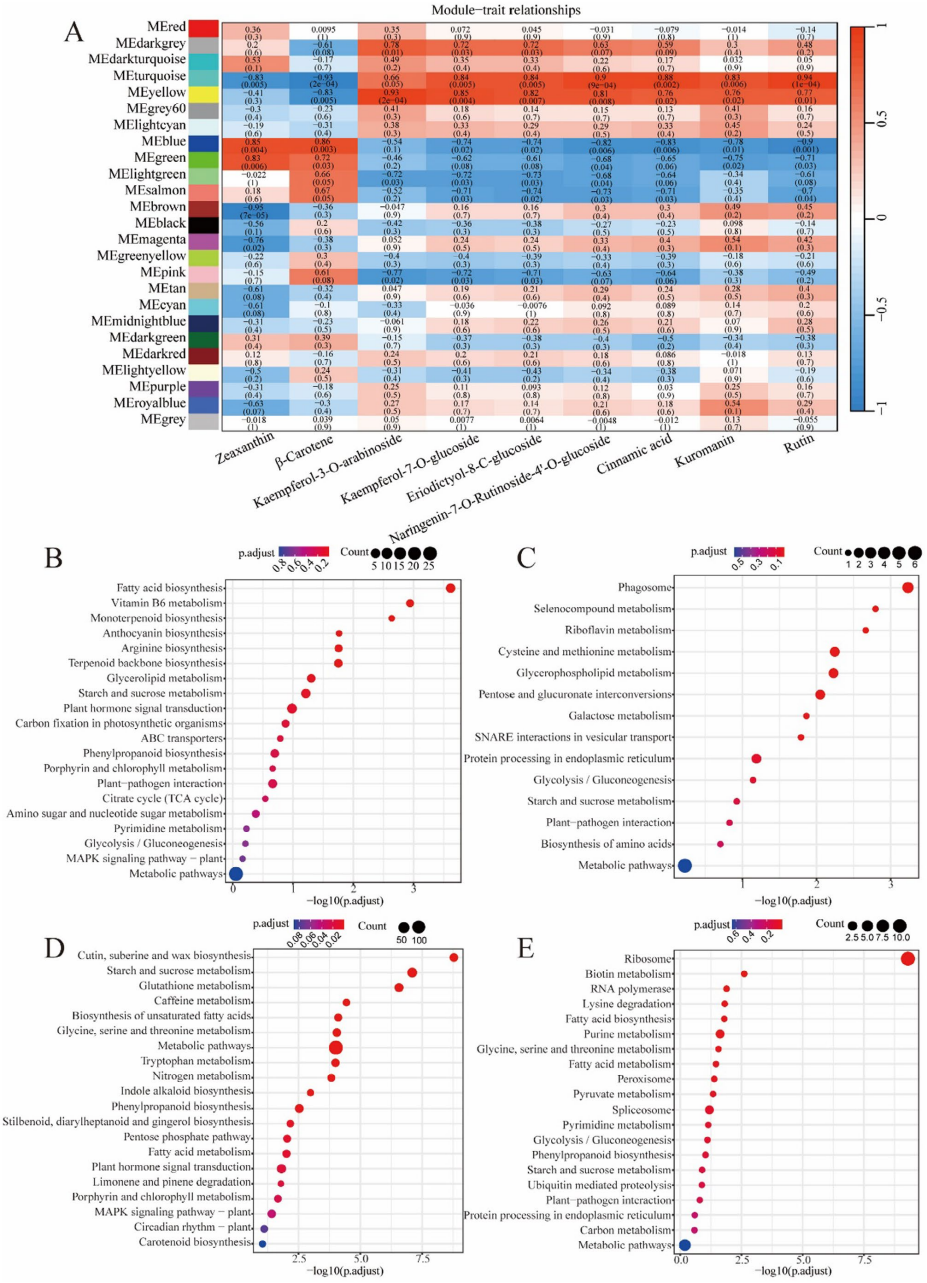
Weighted gene co‐expression network analysis (WGCNA) of genes associated with apocarotenoid and flavonoid, and KEGG analysis of the genes taken from the intersection of four key models and common DEGs. (A) Module‐metabolite association (each row corresponds to a module, and each column represents a specific metabolite. The color of each cell at the row‐column intersection indicates the correlation coefficient between a module and the metabolite). (B) KEGG analysis of blue module. (C) KEGG analysis of green module. (D) KEGG analysis of turquoise module. (E) KEGG analysis of yellow module.

In order to further identify genes associated with flavonoids and apocarotenoids from a comprehensive network perspective, a WGCNA was performed. A total of 25 unique gene modules were discerned from the coexpression patterns of individual genes. These modules, each colored differently, are depicted as a clustergram and network heatmap, as shown in Figure S3A. According to the “module‐ character” correlation analysis, the blue and green modules showed significant positive correlations with zeaxanthin (*r* ^2^ = 0.85 and 0.83, respectively) and β‐carotene (*r* ^2^ = 0.86 and 0.72, respectively). The turquoise module has a positive correlation with kuromanin (*r* ^2^ = 0.83) and rutin (*r* ^2^ = 0.94). The yellow module displayed significant positive correlations with kaemperol‐3‐O‐arabinoside (*r* ^2^ = 0.93), kaemperol‐7‐O‐glucoside (*r* ^2^ = 0.85), eriodictyol‐8‐C‐glucoside (*r* ^2^ = 0.82), naringenin‐7‐O‐rutinoside‐4′‐oglucoside (*r* ^2^ = 0.81), cinnamic acid (*r* ^2^ = 0.76), kuromanin (*r* ^2^ = 0.76), and rutin (*r* ^2^ = 0.77) (Figure 6A). The correlation between gene expression levels and module eigengenes indicates the interconnectivity among genes within each module. A correlation coefficient closer to 1 signifies a stronger association among the genes in the module. Our analysis focused on the genes within modules that exhibited significant correlations with metabolites. The findings revealed that these genes displayed markedly different expression patterns and were highly expressed, with correlation coefficients exceeding 0.70, as illustrated in Figure S4. In conclusion, the blue and green modules demonstrated a strong positive correlation with apocarotenoid biosynthesis, while the turquoise and yellow modules showed a significant positive correlation with flavonoid biosynthesis. The genes of interest were derived from the intersection of these four key models and the 2387 common DEGs presented in Figure S3B. To further determine the function of the genes in blue and green modules related to apocarotenoid biosynthesis, KEGG and GO analyses were performed. The KEGG enrichment analysis showed that monoterpenoid biosynthesis, starch and sucrose metabolism, plant hormone signal transduction, and ABC transporters were highly enriched in the blue module. Notably, this module also exhibited significant enrichment in fatty acid biosynthesis and vitamin B6 metabolism pathways, suggesting potential roles in supporting saffron stigma development (Figure 6B). In the green module, genes were primarily linked to the synthesis and metabolism of primary metabolites (Figure 6C). The GO analysis results for the blue and green modules are shown in Figure S3C,D. The analysis revealed that the blue module is highly enriched in genes associated with responses to diverse environmental stimuli, including hydrogen peroxide, copper ions, flooding, carbon dioxide, and red or far‐red light. This enrichment pattern is consistent with our unpublished experimental observations, which demonstrated a significant increase in crocin content when saffron corms were exposed to red light. However, no noticeable differences in the appearance of the stigmas were observed through visual inspection (Figure S5). Meanwhile, the green module is significantly enriched in pathways related to metabolic substrate and energy supply, cell growth and development, and signal transduction.

The turquoise and yellow gene modules exhibited a positive correlation with the biosynthesis of flavonoids. KEGG enrichment analysis indicated that the turquoise module's genes were substantially involved in the synthesis and metabolism of both primary and secondary metabolites. Moreover, this module was rich in genes associated with plant hormone signaling, porphyrin and chlorophyll metabolism, as well as the MAPK signaling pathway. The yellow module, on the other hand, was characterized by a significant enrichment of genes in starch and sucrose metabolic processes and plant‐pathogen interaction pathways. (Figure 6D,E). In the GO analysis, terms of pigment metabolism, environmental response, as well as cell–cell signaling transduction were observed to be enriched in turquoise module. The DEGs in yellow module were significantly enriched in protein metabolism and cell morphogenesis (Figure S3E,F).

To further determine the relationship between genes within the module and to screen for the hub genes (highly connected genes), a correlation network was constructed (Figure S6). In the blue and green modules, which are positively correlated with apocarotenoid metabolism, we identified *CYP97A3*, *VDE1*, and *HMG3* as key genes directly involved in this process. Additionally, genes that participate in carbohydrate metabolism, such as *G6Pep* and *UGE1*, were also detected. Furthermore, we have identified genes associated with plant hormones (*IBR3*, *CKL2*) and signal transduction pathways (*CALM*, *IQD1*, *TULP5*). The turquoise and yellow modules, showing a positive correlation with flavonoid biosynthesis, have been found to contain genes crucial for this metabolic process. Specifically, *DAHPS2*, a gene directly implicated in flavonoid biosynthesis, plant hormone‐related genes such as *ARF15*, and transcription factors (TFs) such as *MSI4*, *ERF060*, and *NFYA9* have been identified as key regulatory hubs. These genes, due to their central position in the network, are likely to play a significant role in modulating the flavonoid biosynthesis pathway within the stigmas of saffron.

## 4 Discussion

### 4.1 Analysis of Apocarotenoid Biosynthesis Pathway During Saffron Stigma Development

Apocarotenoids exist in the form of volatile and soluble compounds. Crocins are glycosylated derivatives of the apocarotenoid. In addition to being a water‐soluble pigment, crocins are also powerful free radical quenchers, which are linked to their extensive health benefits (Georgiadou et al. 2012; Nam et al. 2010). Crocins are mainly biosynthesized by saffron, which accumulates at high levels during the development of the stigma, conferring a characteristic dark red coloration to the stigma (Moraga et al. 2009). Moreover, picrocrocin, a monoterpene glucoside, is the precursor to safranal, which is the predominant volatile oil component that imparts the characteristic fragrance to saffron (Ahrazem et al. 2016). Consequently, investigating the complex relationships between metabolites and genes within the apocarotenoid biosynthetic pathway is vital, particularly as the saffron stigma develops.

The biosynthesis of crocins necessitates intricate coordination among multiple pathways, including the upstream methylerythritol phosphate (MEP) pathway, the midstream carotenoid biosynthetic pathway, and the downstream crocin biosynthetic pathway (Baba and Ashraf 2016; Ji et al. 2017). Our study revealed remarkable differences in the accumulation of crocins, crocetins, and other apocarotenoids throughout stigma development. Consistent with the findings of Moraga et al. (2009), we observed the highest levels of crocetins and crocins in the red stage (S1 and S2, respectively), accompanied by a corresponding decrease in the intermediate metabolites such as β‐carotene and crocetin dialdehyde. Furthermore, the expression patterns of important genes that are involved in crocin biosynthesis, such as *LCYB, BCH, LUT5*, and *CCD2*, showed a concordant trend, which aligns with earlier research demonstrating that *CCD2* expression peaks during the orange stigma period (Frusciante et al. 2014). This observation suggests that the biosynthesis of crocins is particularly active during the initial phases of saffron stigma development. Besides, the levels of picrocrocin reached the highest levels at anthesis (S3), which was also observed by Moraga et al. (2009). Hence, we hypothesize that the modulation of key genes responsible for crocins biosynthesis in the red stage of stigma development could further enhance the accumulation of crocins.

Interestingly, the ABA content was observed to peak precisely at anthesis (S3). Simultaneously, the genes involved in ABA biosynthesis, such as *ZEP* and *NCED*, also showed peak expression levels during this identical stage. Due to the accumulation of zeaxanthin, the enhanced expression of these genes led to a substantial increase in the biosynthesis of ABA. It was speculated that ABA accumulates to its maximum levels during anthesis, playing a crucial role in various physiological processes within plants, such as senescence and growth inhibition. Since ABA biosynthesis is a metabolic branch that originates from lycopene, it could potentially impact the yield of crocins. Therefore, regulating the biosynthesis of ABA is of great importance. It is necessary to balance the yield of crocins with the need to ensure the normal blooming of flowers. Subsequent studies have revealed that techniques like hydroponics can effectively lower the levels of ABA in the stigmas and markedly increase the concentration of crocins (data unpublished). This further confirms that modulating the metabolic pathways of ABA has a direct impact on crocins production.

CCD1 and CCD7 represent separate subfamily lines within the CCD enzyme family. Notably, CCD7 is instrumental in the pathway for strigolactone biosynthesis (Alder et al. 2012), while enzymes belonging to the CCD1 subfamily exhibit a broad range of cleavage activities on various carotenoids at diverse positions (9,10; 9,10,9′,10′; 5,6,5′,6′; or 7,8,7′,8′) (Ilg et al. 2009; Vogel et al. 2008). In this research, *CCD1* and *CCD7* genes were observed to be expressed most abundantly at S0, suggesting that strigolactones and dihydro‐β‐ionone may accumulate to their highest levels during the early stages of stigma development. The heatmap clearly indicates a gradual decrease in dihydro‐β‐ionone levels throughout the stigma's development. However, it is unfortunate that strigolactones were not detectable in our metabolomics data. Recently, strigolactones have been reported to promote meristem maturation and flower development (Visentin et al. 2024); therefore, making it plausible that they are biosynthesized and accumulate in the earlier stages.

### 4.2 Analysis of Flavonoid Biosynthesis Pathway During Saffron Stigma Development

Flavonoids are crucial secondary metabolites abundantly present in plants, fruits, and seeds, playing vital roles in plant growth and stress resistance. A multitude of research has shown that flavonoids exhibit a broad spectrum of health advantages owing to their diverse bioactivities, which include anti‐inflammatory, anticarcinogenic, anti‐aging, cardiovascular, neuroprotective, immunoregulatory, anti‐diabetic, antibacterial, antiparasitic, and antiviral effects (Fraga et al. 2019; Jucá et al. 2020; Saini et al. 2017). As a result, a growing consumer awareness is emerging about the natural flavonoid content in plants and the health benefits it confers. Studies indicate that saffron's dried stigma tissue contains relatively high levels of these flavonoids (Straubinger et al. 1997; Tarantilis et al. 1995). There is mounting evidence suggesting that flavonoids possess high antioxidant capacity and may serve as potential health‐promoting components in saffron (Rahardhian and Suharsanti 2020). However, there are few studies on the accumulation and biosynthesis pathway of flavonoids at different stigma developmental stages of saffron. In general, flavonoids can be categorized into six major subgroups: chalcones, flavones, flavonols, flavanones, anthocyanins, and isoflavonoids. The heatmap of anthocyanins, flavones, and flavonols revealed that most metabolites were upregulated in stage 1 (Figure 4A–C), indicating that flavonoid biosynthesis is highly active in the initial phase of stigma development. To confirm this speculation, the biosynthesis pathways of flavonoids were mapped, and the transcription of structural genes was integrated (Figure 4D). PAL, C4H, and 4CL are enzymes that catalyze the initial reactions in the flavonoid biosynthetic pathway, laying the foundation for the subsequent reactions (Falcone Ferreyra et al. 2012). Under the enzymatic action of PAL, C4H, and 4CL, phenylalanine is initially transformed into 4‐coumaric acid CoA. Subsequently, 4‐coumaric acid CoA is transformed into the pivotal metabolite naringenin, facilitated by the action of CHS and CHI enzymes. Catalyzed by different enzymes, naringenin mainly produces three branches: one branch produces flavones (nobiletin, apigenin, and luteolin), the second branch produces flavonols (dihydrokaempferol, kaempferol, quercetin, rutin, and myricetin), and the third branch produces isoflavones (genistein), which is in agreement with the previous findings (Gao et al. 2020; Jiang et al. 2020). We found that the anthocyanins, flavones, and flavonols were significantly accumulated in stage 1, and the expression of genes related to flavonoid biosynthesis, like *PAL*, *C4H*, *4CL*, and *CHS*, showed similar changes, which demonstrated that these genes may play a significant role in the synthesis of flavonoids. These results suggest that the biosynthesis of flavonoid metabolites can be enhanced during the early stage of stigma development, so as to increase the antioxidant value of saffron.

### 4.3 Gene Networks Regulating Apocarotenoid and Flavonoid Metabolism

Sophisticated metabolic processes like apocarotenoid and flavonoid biosynthesis are governed by the interplay of multiple genes and cannot be attributed to the action of a single gene. The regulation of genes within these pathways typically occurs through synchronized expression patterns, leading to the frequent use of correlation‐based approaches to uncover gene networks. We identified 25 gene modules/networks with WGCNA, among which 2 exhibited a strong correlation with apocarotenoid metabolism and another 2 were significantly associated with flavonoid biosynthesis.

In the present study, modules correlation with apocarotenoid and flavonoid biosynthesis were notably enriched in pathways related to starch and sucrose metabolism, as well as in plant hormone signal transduction (Figure 6B–E). Sugars not only act as carbon skeletons that supply substrates for the development of sink tissues, but also function as signaling molecules or stimuli that affect metabolic processes and regulate the expression of relevant genes (Hellmann and Smeekens 2014). Lv et al. (2022) demonstrated that the sugar signal initiates the transcription of the hormone‐related transcription factor *CsERF1B‐like*, which subsequently enhances the biosynthesis of flavonoids. Besides, plant hormones can not only regulate plant development, metabolism, aging, and other physiological processes by affecting nucleic acids, proteins, and enzymes in plants, but also regulate the biosynthesis of flavonoids, terpenoids, alkaloids, and other secondary metabolites in plants, which are crucial at multiple stages throughout plant growth and development (Brunetti et al. 2018; Yang et al. 2022). Lee et al. (2011) found that the supplementation of indole acetic acid (IAA) not only promoted the growth of callus tissue and the formation of adventitious roots in mulberry, but also boosted the levels of rutin. Similarly, the use of indole butyric acid (IBA) and naphthaleneacetic acid (NAA) was found to enhance saponin content in ginseng cell cultures (Jeong et al. 2009). In this study, key genes that are central to the metabolic pathways of flavonoids and apocarotenoids were identified. Specifically, *G6Pep*, *UGE1*, and *SBEI*, which participate in carbohydrate metabolism, as well as *IBR3*, *CKL2*, and *ARF15*, associated with plant hormone signaling, have been recognized as hub genes (Figure S6). These genes are expected to have a significant impact on the production of these secondary metabolites. Furthermore, our study found that the biosynthesis of apocarotenoids and flavonoids was enriched in responses to different environmental cues, such as red light and flooding, suggesting that abiotic stress can influence metabolite biosynthesis by altering the expression of related genes. These secondary metabolites are crucial for plants to cope with adverse conditions, defend against attacks, and interact with other organisms (Zhan et al. 2022). Both biotic and abiotic stressors can substantially influence the synthesis, accumulation, and profile of secondary metabolites within plants. Conversely, secondary metabolites serve as a crucial component of defense‐related signaling pathways and can also trigger the widespread expression of defense‐related genes. For instance, Llorente et al. (2016) observed a significant increase in lycopene and β‐carotene content in tomato fruits treated with a high red/far red light ratio, likely attributed to enhanced *PSY* expression. Similarly, Ma et al. (2012) treated in vitro cultured citrus peel with red light LED, and it was found that the red light could effectively upregulate the expression of enzyme genes such as *PSY*, *LCYB1*, and *LCYB2*, resulting in a notable increase in β‐cryptoxanthin content in the citrus peels (Alrifai et al. 2021). Flooding stress suppresses genes related to cytokinin catabolism and general defense response in tomato plants, and enhances the expression of genes involved in ethylene biosynthesis, anthocyanin biosynthesis, and gibberellin biosynthesis (Ngumbi et al. 2022). Therefore, we speculate that the application of special abiotic stress could potentially aid in breeding superior varieties of saffron and enhancing the production of high‐value secondary metabolites for human benefit.

## 5 Conclusions

Our comprehensive analysis of the apocarotenoid and flavonoid biosynthesis pathways during saffron stigma development has provided valuable insights into the complex regulatory mechanisms underlying the production of these important secondary metabolites. We have identified key genes and metabolites involved in these pathways, highlighting their dynamic changes across different developmental stages. The findings suggest that the biosynthesis of crocins and flavonoids is particularly active during the early stages of stigma development, with significant accumulation of these compounds during specific periods.

Additionally, our gene network analysis using WGCNA has revealed that the biosynthesis of these metabolites is intricately linked to carbohydrate metabolism and plant hormone signaling, emphasizing the importance of these pathways in regulating secondary metabolite production. However, it is important to note that our WGCNA analysis was conducted with a relatively small sample size (*n* = 9), which is below the typical recommendations for WGCNA (≥ 8 independent samples or ≥ 15 samples including biological replicates). This limitation may affect the stability of the co‐expression network and potentially introduce false‐positive modules. Therefore, future studies with larger sample sizes are needed to validate and expand upon our findings.

Furthermore, the significant enrichment in responses to environmental cues, such as red light and flooding, indicates that abiotic stress can significantly influence the biosynthesis of apocarotenoids and flavonoids. These results not only enhance our understanding of the metabolic regulation in saffron but also provide a foundation for future studies aimed at optimizing the production of high‐value secondary metabolites through targeted genetic and environmental interventions.

## Author Contributions

**Jing Chen:** conceptualization (lead), formal analysis (lead), writing – review and editing (lead). **Xuting Xu:** data curation (lead), investigation (lead), visualization (lead). **Shuhui Yang:** methodology (equal), writing – original draft (lead). **Xiaodong Qian:** project administration (lead). **Yuanyuan Tao:** data curation (equal). **Guifen Zhou:** validation (equal). **Jing Li:** validation (equal). **Limin Xu:** data curation (equal). **Liqin Li:** funding acquisition (lead), resources (lead), supervision (lead).

## Ethics Statement

The authors have nothing to report.

## Consent

All authors are aware of and agree to submit the article to this journal.

## Conflicts of Interest

The authors declare no conflicts of interest.

## Supporting information


Data S1.

Data S2.

## Data Availability

Data available on request from the authors.
