# Supplementary material for: Integrative Analyses of Metabolome and Transcriptome Reveals Apocarotenoid and Flavonoid Biosynthesis During Saffron ( Crocus sativus L.) Stigmas Development
Source: Food Sci Nutr. 2025 Aug 1;13(8):e70712. doi: 10.1002/fsn3.70712 (PMC12317191; doi:10.1002/fsn3.70712)
Supplement: Supplementary file 1 — Data S1. [file FSN3-13-e70712-s002.docx]

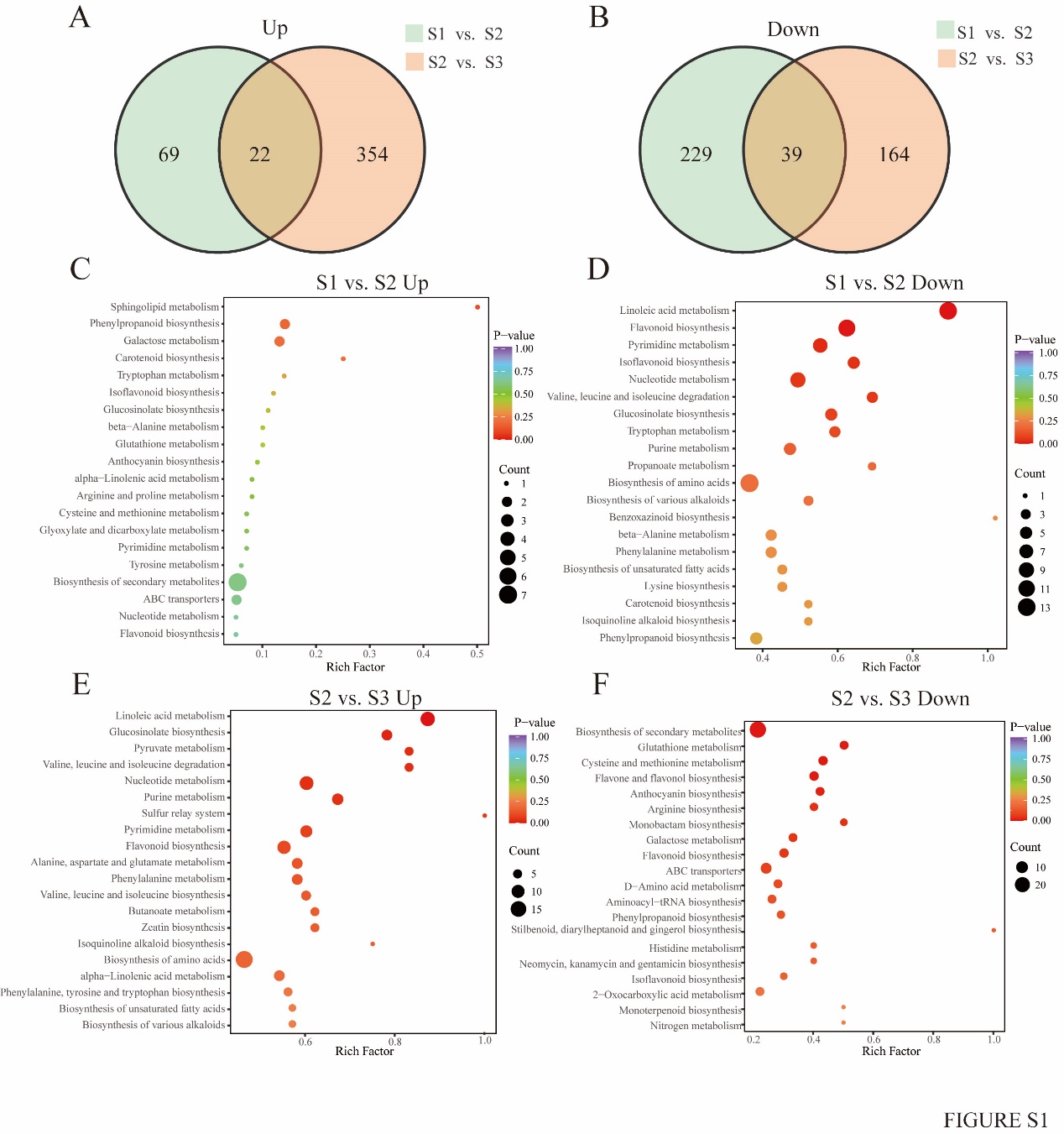


**FIGURE S1 |** Summary of differentially accumulated metabolites (DAMs) upregulated and downregulated in the comparisons between S1 vs. S2 and S2 vs. S3 of saffron stigmas development. (A) Venn diagrams of DAMs upregulated. (B) Venn diagrams of DAMs downregulated. (C) KEGG pathway analysis of DAMs upregulated in the comparisons between S1 vs. S2. (D) KEGG pathway analysis of DAMs downregulated in the comparisons between S1 vs. S2. (E) KEGG pathway analysis of DAMs upregulated in the comparisons between S2 vs. S3. (F) KEGG pathway analysis of DAMs downregulated in the comparisons between S2 vs. S3.


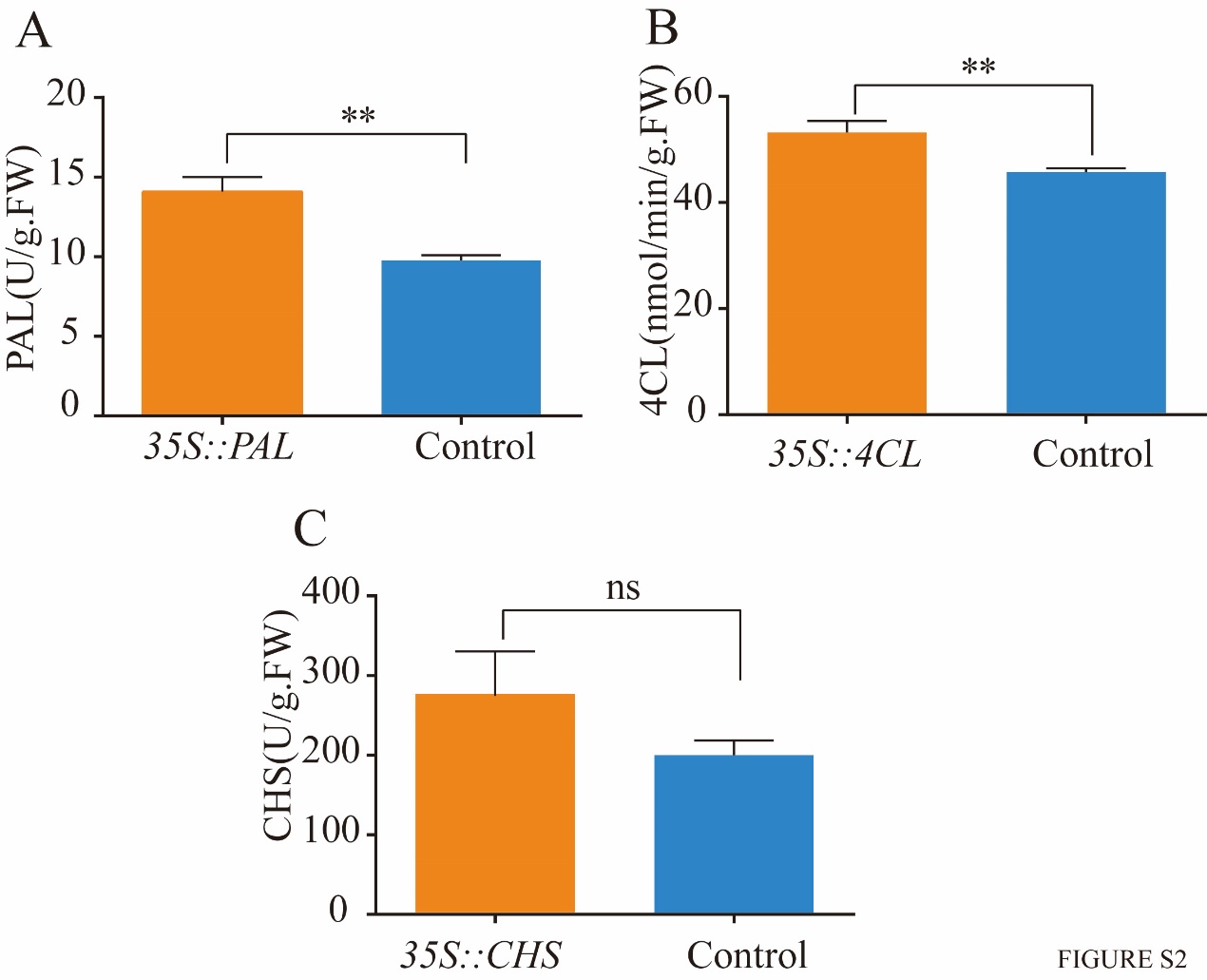


**FIGURE S2 |** Enzyme activity assays of key flavonoid biosynthesis genes after transient expression in tobacco. (A) Transient expression of phenylalanine ammonia-lyase (*PAL*) in tobacco leaves; (B) Transient expression of 4-coumarate: CoA ligase (*4CL*) in tobacco leaves; (C) Transient expression of chalcone synthase (*CHS*) in tobacco leaves. Statistical significance was determined using independent samples t-test. **P < 0.01.


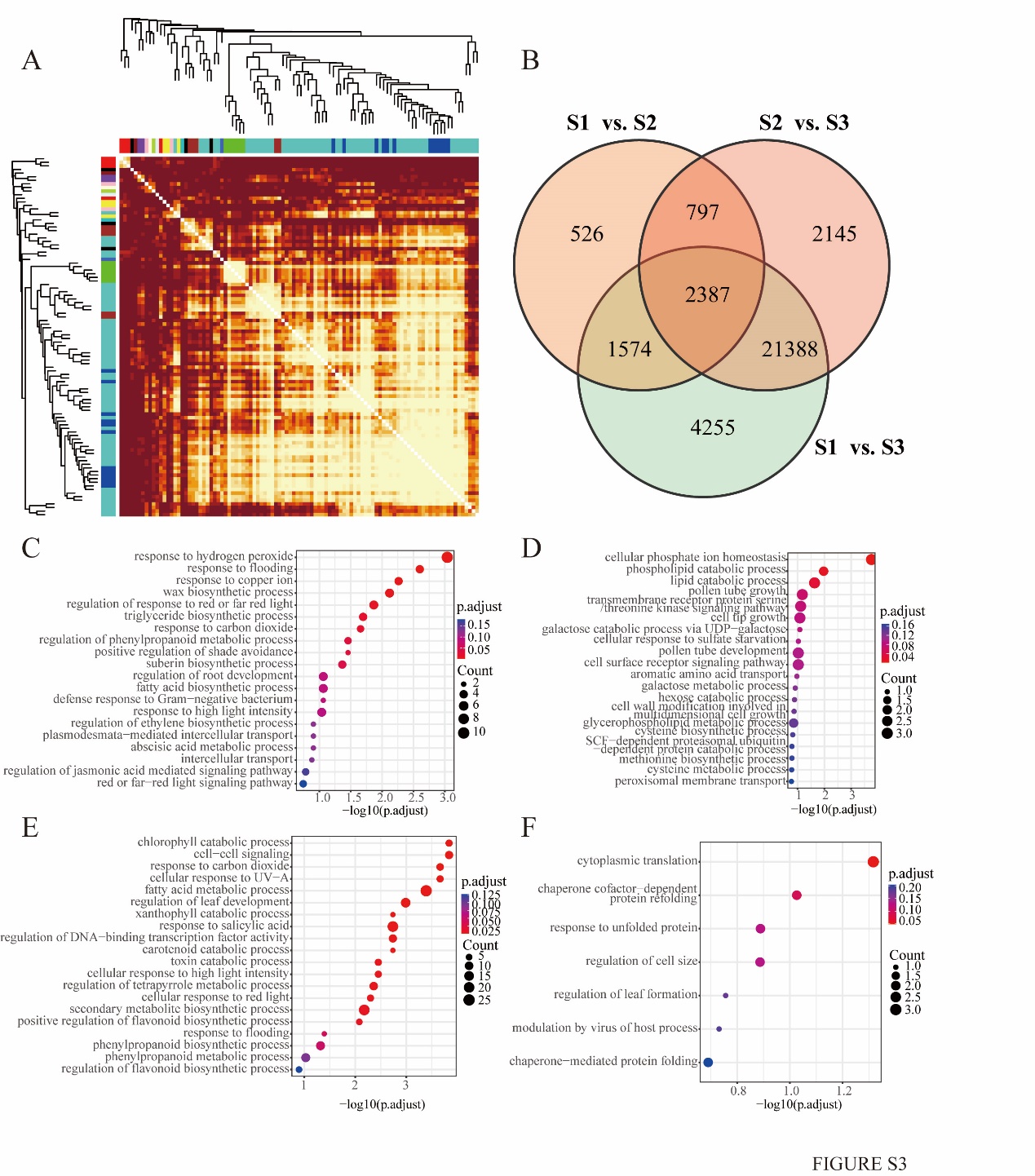
 **FIGURE S3 |** Coexpression modules and functional enrichment of differentially expressed genes (DEGs) during saffron stigmas development. (A) Cluster dendrogram and network heatmap of genes subjected to coexpression module calculation. (B) Venn diagrams of DEGs in the comparisons between S1 vs. S2, S2 vs. S3 and S1 vs. S3 of saffron stigmas development. (C) Gene Ontology (GO) analysis for the blue module. (D) GO analysis for the green module. (E) GO analysis for the turquoise module. (F) GO analysis for the yellow module.


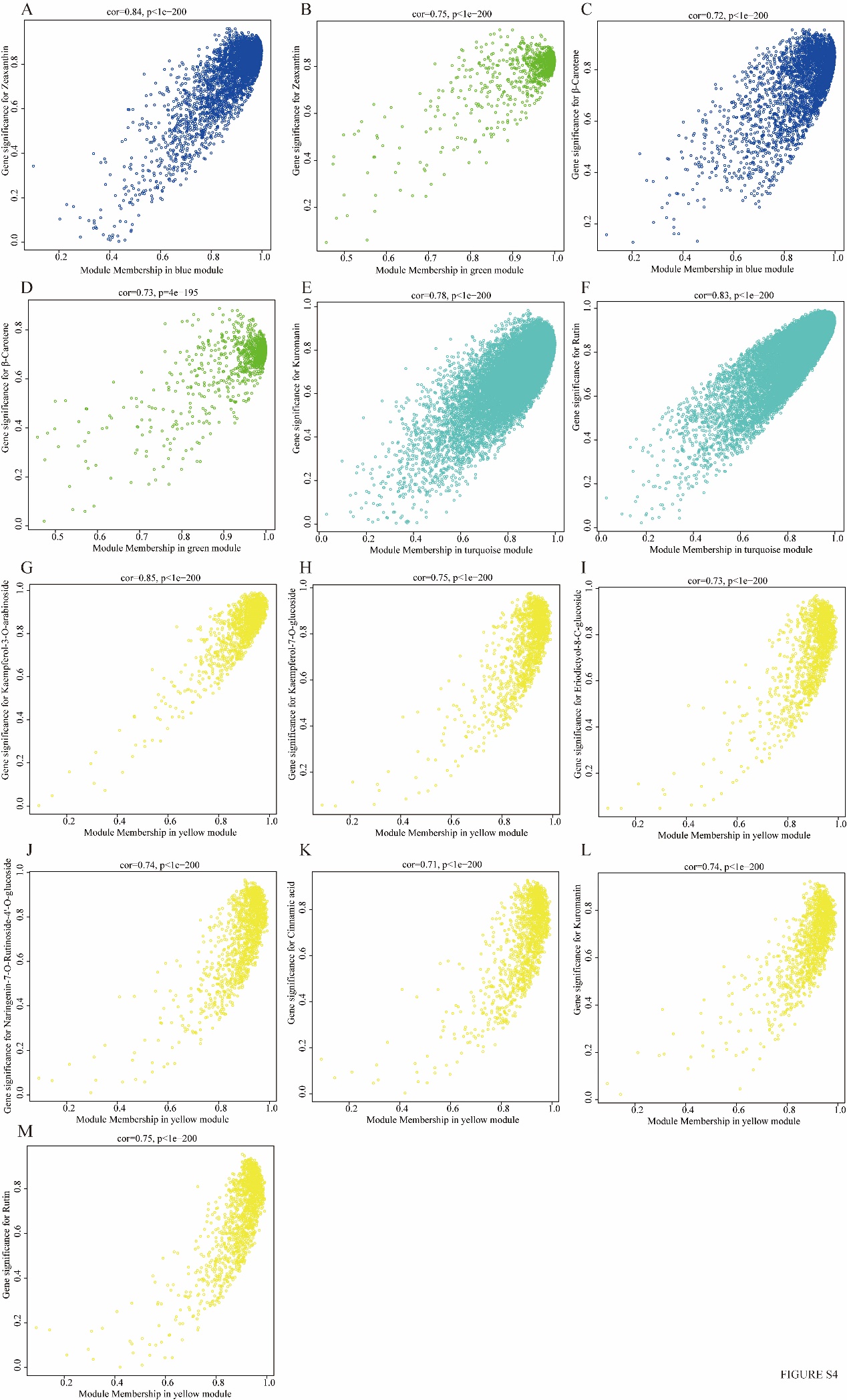


**FIGURE S4 |** Module membership (MM) and gene significance (GS) in selected modules. Each color represents a selected WGCNA module. In each plot, the y-axis represents the GS of a fermentation trait and the x-axis represents the MMs of selected modules that were highly associated with those traits (correction coefficient > 0.7 and p < 0.05)


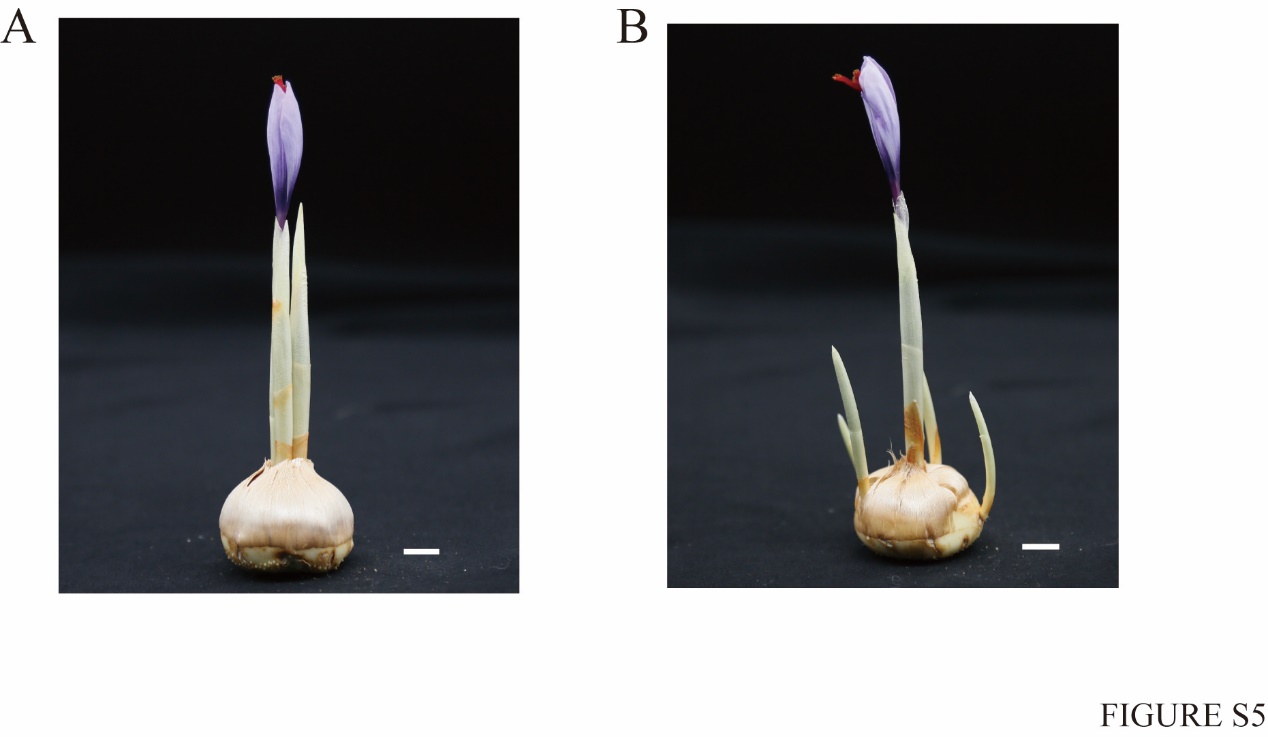


**FIGURE S5 |** Phenotypes of saffron during flowering under red light. (A) Control group. (B) Red light treatment group.


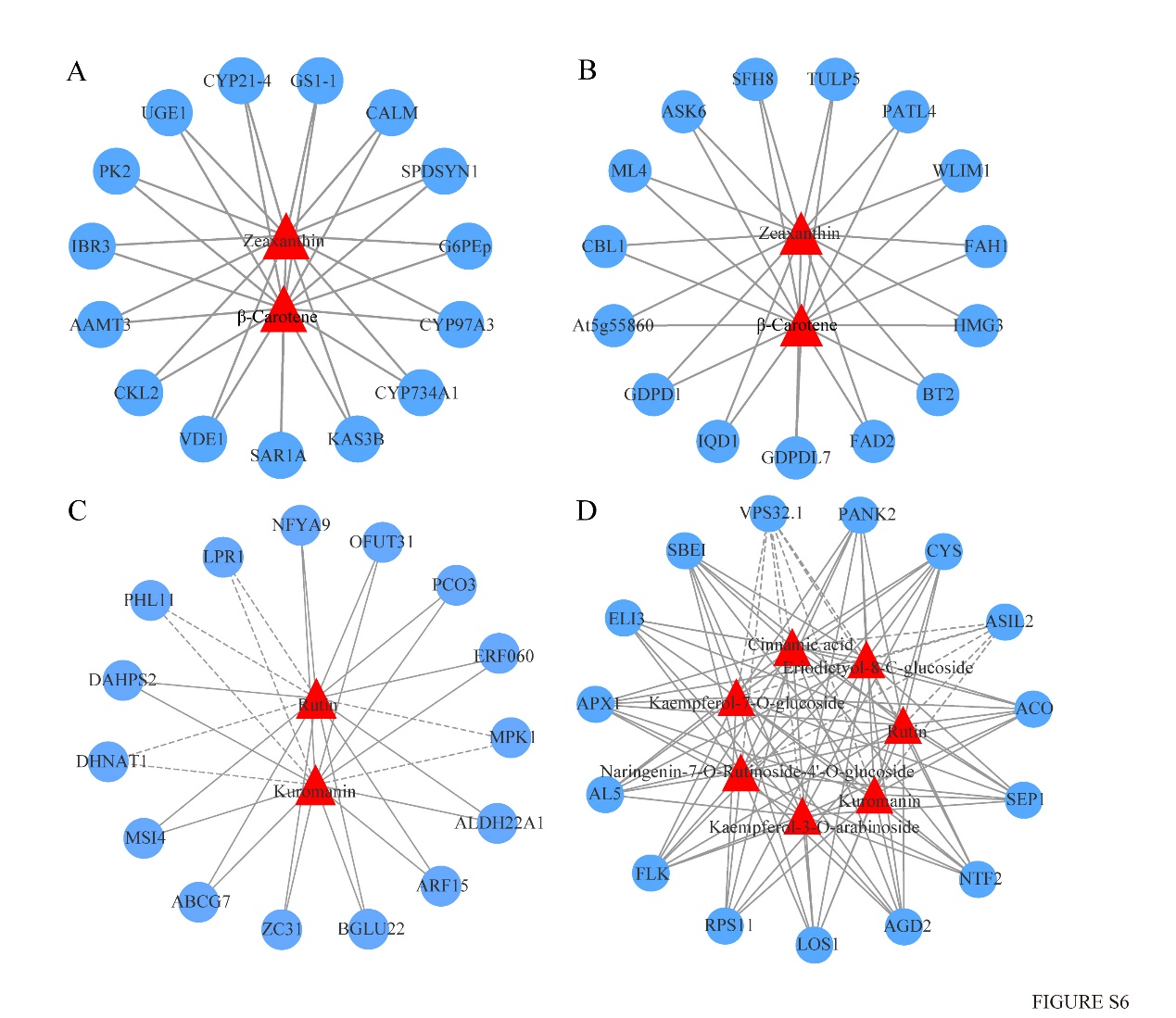


**FIGURE S6 |** The correlation networks of genes in selected modules. Triangles represent metabolites and circles represent hub genes. (A) Brown module. (B) Blue module. (C) Yellow module. (D) Turquoise module.
